# Human Metabolites of *Hamaforton™* (*Hamamelis virginiana L.* Extract) Modulates Fibroblast Extracellular Matrix Components in Response to UV-A Irradiation

**DOI:** 10.3389/fphar.2021.747638

**Published:** 2021-12-17

**Authors:** Fausta Natella, Barbara Guantario, Roberto Ambra, Giulia Ranaldi, Federica Intorre, Carolina Burki, Raffaella Canali

**Affiliations:** ^1^ Research Centre for Food and Nutrition, CREA-Consiglio per la Ricerca in Agricoltura e l’Analisi dell’Economia Agraria, Rome, Italy; ^2^ Horphag Research, Geneva, Switzerland

**Keywords:** hamamelitannins, human study, fibroblast, UV-A, extracellular matrix, *Hamamelis virginiana*, gene expression

## Abstract

*Hamamelis virginiana L.* a rich source of both condensed and hydrolyzable tannins, utilized to treat dermatological disorders. Since no experimental and clinical data is available for its use as oral formulation in skin related disorders, the purpose of this study was to investigate the effects of *Hamaforton™* (*Hamamelis virginiana* extract) metabolites on gene dysregulation induced by ultraviolet A radiation in cultured human dermal fibroblasts. A combination of *in vivo* and *ex vivo* experimental designs has been exploited in order to take into account the polyphenol metabolic transformation that occurs in humans. 12 healthy volunteers received either a capsule of *Hamaforton™* or a placebo in a randomized, blinded crossover trial. After *Hamaforton™* ingestion, the kinetic of appearance of galloyl derivatives was measured in plasma. Then, in the *ex vivo* experiment, the serum isolated after supplementation was used as a source of *Hamaforton™* metabolites to enrich the culture medium of dermal fibroblasts exposed to ultraviolet A radiation. Three different gallic acid metabolites (4-O-methyl gallic acid, 4-O-methyl gallic acid sulphate and trimethyl gallic acid glucuronide) were identified in volunteer plasma. While, ultraviolet A irradiation of dermal fibroblasts affected the expression of extracellular matrix genes, the presence of *Hamaforton™* metabolites in the culture media did not affect the expression of most of those genes. However, the activation of the expression of 10 different genes involved in repair processes for the maintenance of skin integrity, suggest that the metabolites can play a role in damage recovery. To our knowledge, this is the first study that demonstrates the bioavailability of *Hamaforton™* phenolic compounds, and the effects of its metabolites on cultured dermal fibroblast response to ultraviolet A irradiation.

## Introduction

*Hamamelis virginiana L.* also known as witch hazel, a rich source of both condensed and hydrolyzable tannins, is a shrub that belongs to the Hamamelidaceae family. Originating from the eastern regions of the United States, where the plant is among the most popular herbal products, witch hazel is now also grown in Europe. Different formulations of *Hamamelis v.* leaves, bark and twigs, commercially available as extracts, tinctures, distillates, creams and lotions, are utilized for dermatological disorders. They are used for their astringent and antiphlogistic properties as anti-inflammatory agents for skin disorders, to promote healing and alleviate sunburn, skin or mucosal irritation, phlebitis, varicose veins, ulcers of varicose veins and haemorrhoids (Reuter et al., 2010).

Despite its extensive use as herbal remedy, there are very few studies that have evaluated the background of its effectiveness. *In vitro* investigations evaluated the anti-tumoral, antioxidant and anti-inflammatory activity of witch hazel extracts (Lizárraga et al., 2008; Thring et al., 2009; Thring et al., 2011; Sánchez-Tena et al., 2012), while *in vivo* human approaches are limited to the study on the effects of topical treatments against ultraviolet radiation-induced erythema (Hughes-Formella et al., 2002). However, no experimental and clinical data is available for the use of witch hazel extract as oral formulation in skin-related problems. Therefore, a preclinical model (Natella et al., 2014) of *ex vivo* cultured dermal fibroblast incubated with human sera after *Hamamelis v.* extract supplementation was used to study the effects of *Hamamelis v.* metabolites on cellular damage induced by ultraviolet radiation-A irradiation.

It is assumed that around 80% of skin aging is caused by UV exposure and studies indicate that the photoaging process affects extracellular matrix (ECM) components (Freitas-Rodríguez et al., 2017). Sunlight is composed primarily of UV-A (90–95%) and UV-B (5–10%) energy, with UV-A having the longest wavelength (315–400 nm) (Amaro-Ortiz et al., 2014). UV-A rays are lower in energy, but they are at least 20 times more abundant. Unlike UV-B radiation, UV-A radiation is partly absorbed by the epidermis, but due to its high penetration properties, 20–30% of it can reach deeper parts of the skin and affect the dermal compartment (Battie et al., 2014). Photoaged skin is characterized by loss of skin tone dryness, irregular pigmentation and deep wrinkles formation. *Hamamelis v.* contains hydroxycinnamic acids, flavonoids, condensed tannins and hydrolyzable tannins such as hamamelitannin and pentagalloylglucose, whose biological activities were partially elucidated (Duckstein and Stintzing, 2011). The chemical structure of these molecules is modified following intestinal absorption by the detoxifying phase II metabolism yielding more hydrophilic and less reactive conjugated compounds (Serreli and Deiana, 2019).

The aim of this study was to evaluate the bioavailability of *Hamaforton™* (*Hamamelis virginiana* extract) components after human supplementation as well as to utilize those metabolites to enrich the cell culture media of human dermal fibroblast in order to study their biological activity.

## Materials and Methods

### Analysis of *Hamaforton™* Capsule

The capsules *Hamaforton™*, a proprietary *Hamamelis v.* extract, were provided by Horphag Research. Each capsule contains 300 mg of the extract of the aerial parts of the plant. To study the phenolic compound composition, one *Hamaforton™* capsule was extracted with 50 ml of MeOH:H_2_O 80:20 (v/v) under continuous agitation for 18 h. Subsequently, the extract was centrifuged for 10 min at 2,000 g and 4°C. The supernatant was filtered through 0.45 and 0.20 µm polyvinylidene fluoride (PVDF) filter. Initially, we performed a qualitative analysis in which extracts were infused directly into the electrospray ionization (ESI) source of the mass spectrometer and spectra were acquired using negative ion mode. The identity of the *Hamaforton™* compounds was verified by the comparison of the mass spectra recorded for each compound with those of standards and/or with those reported in literature (Duckstein and Stintzing, 2011). Then, we performed a quantitative analysis by using tandem mass spectrometry (MS/MS) coupled with High Performance Liquid Chromatography (HPLC) and using a Multiple Reaction Monitoring (MRM) method; when authentic standard was not available, compounds were quantified using the standard curve of a chemically related compound.

### *In Vivo* Study

Fourteen, non-smoking, healthy volunteers (7 males and 7 females), aged 28–55 years, with a Body Mass Index (BMI) between 19.5 and 27 kg/m^2^, were enrolled in a double-blind randomized crossover study. Volunteers were instructed to abstain from dietary supplements intake and medications at least 15 days prior the experiments and, in order to avoid diet-introduced phenolic compounds and tannins, to refrain from consuming wine, beer, coffee, tea, fruit juice, fruits and vegetables, nuts, chocolate, olive oil and spices during the 24 h before the experiments. To ensure adherence to dietary instructions, subjects were asked to keep a dietary record prior study participation. The subjects were randomly assigned to receive *Hamaforton™* capsule or placebo; they attended the laboratory on two separate occasions (2 weeks apart) after overnight fast. A venous blood sample was taken at time 0 h (time 0). Immediately after the first blood collection, participants were provided with one capsule of *Hamaforton™* extract or of placebo. Further blood samples were taken at 1, 3, and 5 h (time 1, time 3, time 5) after the supplementation. After sampling, plasma and serum were each separated from blood and stored at −80°C until used. Approval was obtained from the Ethical Committee of ASL Roma 2, Rome, (trial registration n: 133.17; prot. 0065390/2018 of the April 18, 2018). Each subject gave written informed consent for the study. The study was completed by 12 volunteers (6 males and 6 females).

### Metabolite Analysis

Plasma samples (500 µl) were thawed at room temperature and diluted (1:1) with phosphoric acid 4%, spiked with the internal standard syringic acid (0.1 mg/ml) and centrifuged at 16,400 g for 15 min at 4°C. Diluted samples were loaded (600 µl) on a 96 well-SPE OASIS HLB plate, washed with 200 µl of water and 200 µl of 0.2% acetic acid and finally eluted with methanol (60 µl) for HPLC-ESI-MS/MS analysis.

Samples were analysed by an HPLC system interfaced to an Applied Biosystems (Foster City, CA, United States) API3200 Q-Trap instrument. LC analyses were conducted using a system equipped with a binary pump (PerkinElmer, Waltham, MA, United States). Samples were injected (10 µl) into Kinetex column (Phenomenex) (2.6 μm C18 100A, 100 mm × 2.1 mm) and eluted at flow rate of 0.25 ml/min. Mobile phase A was constituted by H_2_O containing 0.1% formic acid while mobile phase B was constituted by acetonitrile containing 0.1% formic acid. Elution was carried out using a gradient commencing at 0% B and changing to 100% B in 37 min. The flow from the chromatograph was injected directly into the ESI source. The MS operated with an electrospray voltage at −4,500 V and with source temperature of 400°C. Nitrogen was used as ion spray (GS1), drying (GS2), and curtain gas at 10, 20, and 10 arbitrary units, respectively. A targeted metabolomics approach was used to search *Hamaforton™* metabolites. The metabolites investigated were chosen from the most representative on the basis of a literature survey (Barnes et al., 2016; Fang et al., 2018); up to 40 compounds related mainly to the metabolism of gallotannin, ellagic acid, quercetin, catechin, kampferol and some phenolic acids (ferulic, coumaric and caffeic acid), were monitored in MRM. The selection was made considering metabolites from different metabolic pathways related to the phenolic compounds contained in the *Hamamelis v.* extract (see Table 1). The declustering potential (DP), collision energy (CE) and entrance potential (EP) were optimized for each metabolite using the parameter of the commercially available standard. Quantification of 4-O-methyl gallic acid was performed with calibration curves of the standard. Parent/product ion pairs (MRM transitions) and MRM condition of identified phenolic metabolites are listed in Supplementary Table S1. Due to lack of standards, the quantification of 4-O-methyl gallic acid sulphate and trimethyl gallic acid glucuronide was tentatively done using the calibration curve of 4-O-methyl gallic acid. Data acquisition and processing were performed using Analyst software 1.5.1.

**TABLE 1:** Phenolic composition of *Hamaforton™* capsule. The analysis was performed with HPLC-MS/MS; results are expressed as mean ± SD.

	Compound	mg/capsule
Galloyl derivatives	Nonagalloyl hexose	tr
	Heptagalloyl hexose^a^	0.04 ± 0.01
	Hexagalloyl hexose (3 isomers)^a^	0.35 ± 0.08
	Pentagalloyl glucose^a^	1.94 ± 0.58
	Tetragalloyl hexose (4 isomers)^a^	0.18 ± 0.05
	Trigalloyl hexose^a^	0.12 ± 0.04
	Monogalloyl hexose^a^	0.21 ± 0.02
	Digalloyl-hamamelose (hamamelitannin)	1.08 ± 0.13
	Ethyl gallate	16.92 ± 2.60
	Ethyl digallate^b^	1.00 ± 0.27
	Methyl gallate	2.21 ± 1.10
	Digallic acid (2 isomers)^a^	2.24 ± 1.01
	Galloyl quinic acid (theogallin)^a^	0.59 ± 0.27
	Gallic acid	10.33 ± 2.68
	Ellagic acid	0.82 ± 0.27
Flavonol derivatives	Kaempferol galloyl hexoside^c^	0.04 ± 0.01
	Kaempferol	1.75 ± 0.56
	Quercetin	1.89 ± 0.45
	Quercetin rutinoside (rutin)	1.27 ± 0.33
	Catechin	0.5 ± 0.06
Phenolic acid derivatives	Coumaril quinic acid (2 isomers)^d^	0.09 ± 0.03
	Chlorogenic acid (2 isomers)	2.3 ± 0.5

a quantified as equivalent of Gallic acid.

b quantified as equivalent of Ethyl gallate.

c quantified as equivalent of Kaempferol.

d quantified as equivalent of Coumaric acid.

Tr, trace.

### *Ex Vivo* Study

#### Cultured Cells and UV-A Irradiation

**FIGURE 1:**
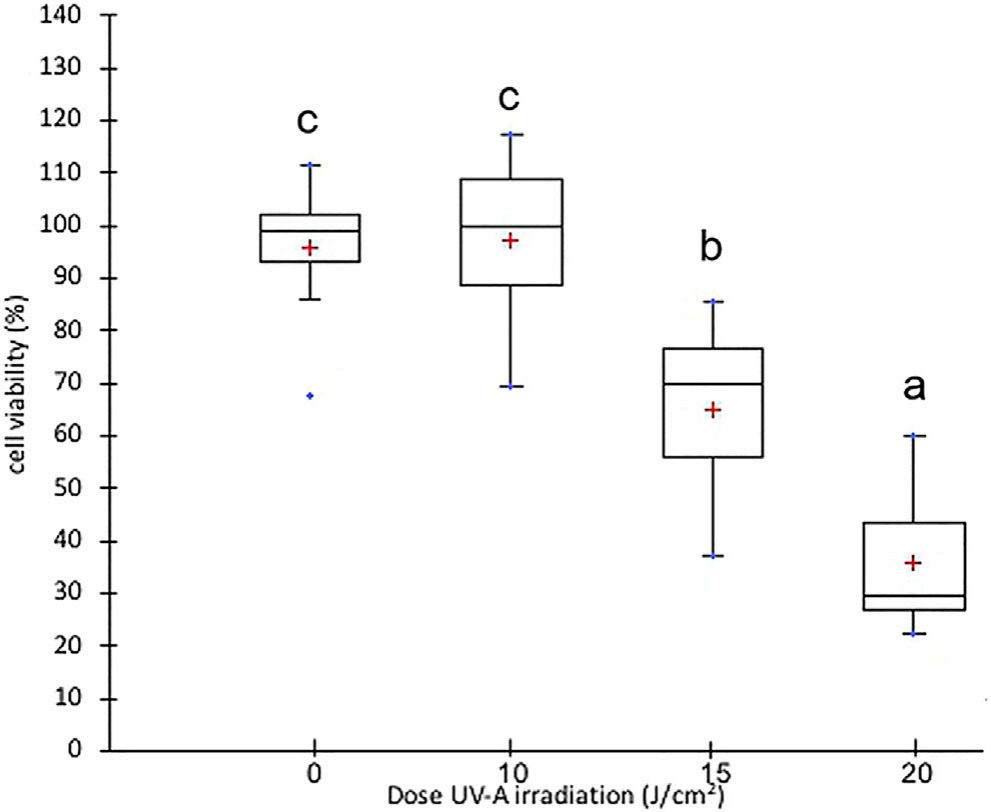
Cell viability in fibroblast irradiated with different doses of UV-A. Box plot representation of cell viability expressed as % of control cells. The red crosses correspond to the means while the central horizontal bars represent the medians. The dots represent the maximum and minimum values. The data were analysed through Welch One-way ANOVA followed by post hoc Tamhanes’ test (*p* < 0.05). Normal distribution and homogeneity of variance were assessed with Shapiro–Wilk and Levene’s tests, respectively, prior to the analysis.

Primary Human adult Dermal Fibroblasts (HDF) were purchased from Sigma Aldrich/European Collection of Authenticated Cell Cultures (ECACC) as cryopreserved vials (Lot 2134). Cells were grown in the fibroblast growth medium (Sigma Aldrich, St Louis, MO, United States) supplemented with 10% inactivated Fetal Bovine Serum (FBS) (Euroclone, Pero, MI, Italy), 2 mM L-glutamine (Corning, New York, United States) and 1% penicillin/streptomycin (Corning, New York, United States), in a 37°C humidified incubator containing 5% CO_2_. For the experiments, cells were cultured in Dulbecco’s Modified Eagles Medium (DMEM, low glucose) (Corning, New York, United States) with 10% inactivated FBS. Cells were treated between passages 3 and 9. Preliminary experiments were carried out to identify optimal UV-A doses for cell experiments. Specifically, cell viability was assessed by MTT cell proliferation assay kit in 96 well plates (Molecular Probes, OR, United States). HDFs were seeded at a density of 1 × 10^4^ cells/well and exposed to UV-A at doses of 10, 15, and 20 J/cm^2^ (Nakyai et al., 2017) (Marionnet et al., 2012) in a crosslinker CL-508 (365 nm, UVITEC Cambridge, UK). During UV-A irradiation, cells were incubated in PBS (Corning, New York, United States); then, cells were further incubated for 24 h in DMEM complete medium to allow time for the activation of damage or repair systems. Control HDF cells were treated similarly, except for UV-A irradiation. Each assay was repeated in triplicates, and the mean was calculated. As shown in Figure 1, we found a large and significant decrease in viability of fibroblasts irradiated with 15 and 20 J/cm^2^ UV-A, while there was no significant alteration of viability in fibroblasts irradiated with 10 J/cm^2^ UV-A, that was therefore used as treatment dose in next experiments.

### HDF Incubation With Human Serum

Preliminary experiments were performed to identify the serum that was able more than others to affect the expression of genes involved in HDF extracellular matrix turnover (i.e. MMP-1, MMP-2, MMP-7, COL1A1, FN1 and Actin B), among those isolated at time 1, time 3 and time 5 h after *Hamaforton™* supplementation. The results demonstrated that *Hamamelis v.* supplementation affected significantly HDF gene expression only with sera isolated after 1 h and 3 h (data not shown). After integrating this data with metabolite results obtained from all subjects, we chose the design using the sera obtained 3 h after supplementation of the subjects to perform following experiments.

### *Ex Vivo* Experimental Design

**FIGURE 2:**
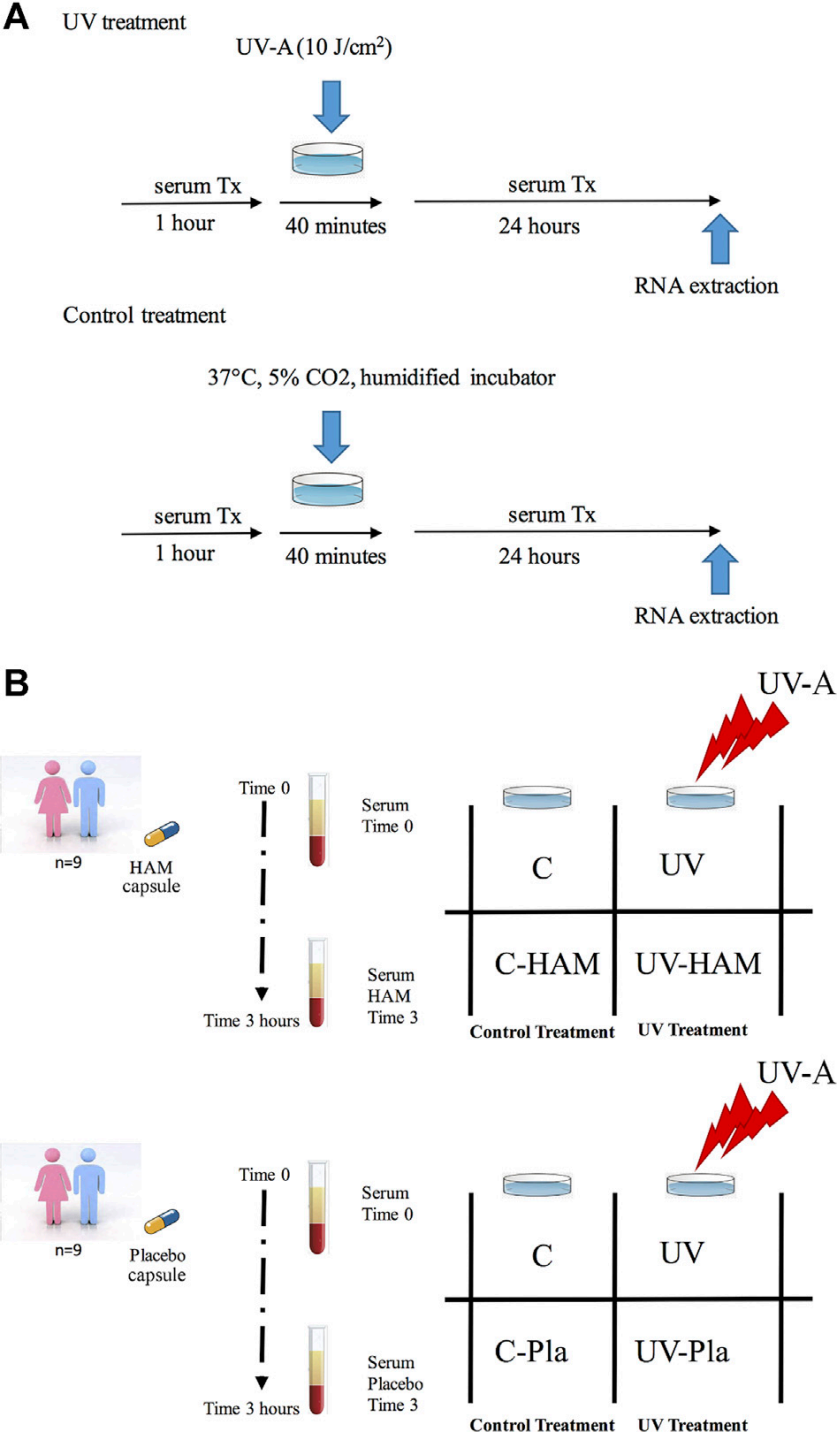
**(A)** Schematic protocol for control and UV treatments; **(B)** Scheme of cellular treatments.

HDF cells at 80–90% confluence (passage number 3–4) were pre-incubated for 1 h in 12-well plates with DMEM containing 10% of time 0 or time 3 serum either isolated from subjects supplemented with *Hamaforton™* or placebo. After this pre-incubation, fibroblasts in PBS solution were exposed to 10 J/cm^2^ UV-A (UV-A treatment) or incubated at 37°C in humidified incubator (control treatment) for 40 min. Then, cells were further incubated for 24 h with fresh DMEM containing the same serum used for the pre-incubation (Figure 2A). At the end of the incubation, cells were harvested and lysed with RLT buffer (RNeasy Plus Mini Kit, Qiagen) and stored at −80°C until use.

Among the 12 subjects that completed the *in vivo* study, we were able to use only the sera isolated from 9 subjects (5 males and 4 females). In fact, the sera from three volunteers caused cellular death. Since the human serum (unlike bovine serum) did not undergo heat inactivation of the complement system before the incubation with cells (in order not to damage *Hamaforton™* metabolites), we cannot exclude the presence of cytolytic components in those sera. For each subject that completed the study, the following cellular treatments were carried out (Figure 2B): C (control cells incubated with time 0 serum); UV (cells incubated with time 0 serum and exposed to UV-A); C-HAM (cells incubated with human serum isolated 3 h after *Hamaforton™* supplementation); UV-HAM (cells incubated with human serum isolated 3 h after *Hamaforton™* supplementation and exposed to UV-A); C-Pla (cells incubated with human serum isolated 3 h after placebo supplementation); UV-Pla (cells incubated with human serum isolated 3 h after placebo supplementation and exposed to UV-A). Thus, 9 biological replicates (coming from the sera isolated from the 9 subjects) were performed for each cellular treatment (no pool was used).

### PCR Arrays

The RT^2^ First Strand Kit (Qiagen, Hilden, Germany) was used for the synthesis of cDNA from RNA, while the expression of genes involved in extracellular matrix pathways and adhesion molecules was carried out with RT^2^ Profiler™ PCR Arrays (Qiagen, Hilden, Germany) using the RT^2^ SYBR Green ROX qPCR Mastermix. The quantification of gene expression was determined by real-time PCR (qPCR) with a 7,500 Fast Real-Time PCR System (Applied Biosystem, Waltham, MA, United States). Data were collected using the 7,500 Software version 2.0.5 and given as threshold cycle (C_t_). C_t_ values for each target and reference gene (GAPDH) were obtained, and their difference was calculated (ΔC_t_). Relative gene expression quantification is expressed as 2^−ΔCt^ for each treatment.

### Data and Statistical Analysis

Normal distribution and homogeneity of variance of all variables were controlled with Shapiro–Wilkinson and Levene’s tests, respectively. The statistical analysis of plasmatic metabolite data was performed using the Kruskal-Wallis or Friedman test depending on the verification of normality and homoschedasticity. The statistical significance of gene expression data was evaluated by paired *t*-test, comparing the effects of sera isolated from each subject before and after *Hamaforton™* (or placebo) supplementation on HDF cells. Differences with *p* values <0.05 were considered significant. Statistical analysis was performed with Microsoft Excel 2011 upgraded with XLSTAT (ver. March 4, 2014).

## Results and Discussion

### Composition of the Capsule

The phenolic composition of *Hamaforton™* capsule, containing 300 mg extract of the aerial parts of the plant *Hamamelis v.*, is shown in Table 1; the main compounds recovered were gallotannins in which galloyl units (up to 9) are bound to polyol carbohydrate. The presence of these compounds, as well as gallic acid and catechins, have been already reported in *Hamamelis v.* extracts of bark, stem and leaf (Wang et al., 2003; González et al., 2010). In the *Hamaforton™* capsule, we also found hydroxycinnamic acids (such as chlorogenic and coumaril quinic acid) and flavonols (such as quercetin and kaempferol), that were reported mainly in leaf extracts of *Hamamelis v.* (Duckstein and Stintzing, 2011).

### Compounds Detected in Plasma Samples

**FIGURE 3:**
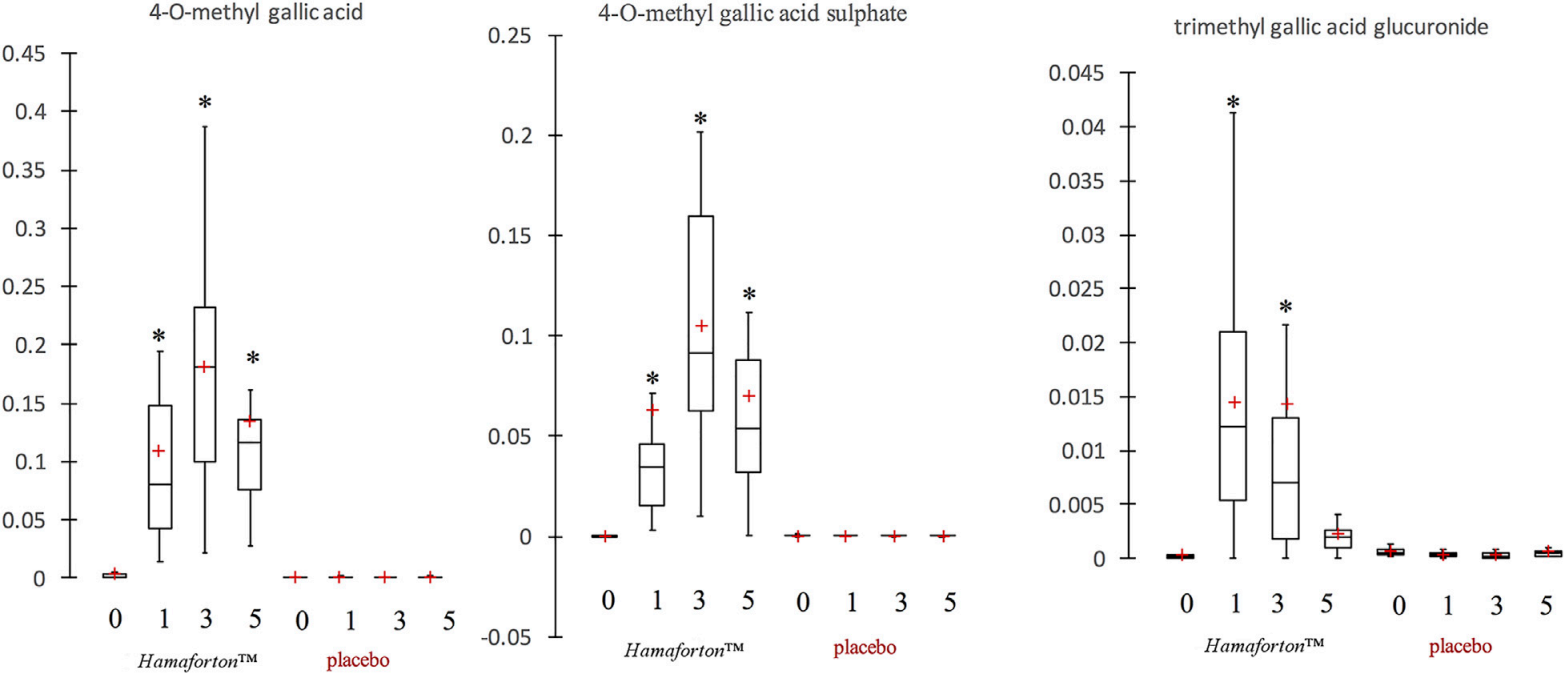
Box plot representation of gallotannin metabolites in plasma (µg/ml) after *Hamaforton™* and placebo capsule ingestion (time 0, time 1, time 3 and time 5 h). The red crosses correspond to the means, while the central horizontal bars represent the medians. The lower and upper limits of the box correspond to the first and third quartiles, respectively. Data were analysed through Kruskal-Wallis and Friedman test as opportune. *indicates significant differences compared to time 0 (*p* < 0.05).

The analysis of phenolic compounds metabolites was performed in plasma samples of the 12 volunteers who ingested a *Hamaforton™* capsule. The HPLC-ESI-MS/MS analysis on human plasma revealed three different gallic acid metabolites (4-O-methyl gallic acid, 4-O-methyl gallic acid sulphate and trimethyl gallic acid glucuronide) appearing after capsule ingestion in all volunteers (Figure 3). None of these metabolites was present in plasma collected at time 0 and before/after placebo ingestion. The maximum concentration reached after capsule ingestion was 0.18 ± 0.11 μg/ml plasma for 4-O-methyl gallic acid, 0.11 ± 0.07 μg/ml plasma for 4-O-methyl gallic acid sulphate and 0.01 ± 0.01 μg/ml for trimethyl gallic acid glucuronide (the last 2 were quantified as 4-O-methyl gallic acid equivalent). The absorption peak occurred at 3 h for 4-O-methyl gallic acid and 4-O-methyl gallic acid sulphate, and at 1 h for trimethyl gallic acid glucuronide. At their peak, the plasma concentration of these quantified metabolites was around 1.5 µM, according to other bioavailability results that showed that the consumption of 10–100 mg of a single phenolic compound rarely exceeded 1 µM of plasmatic maximum concentration (Scalbert and Williamson, 2000). In fact, phenolic bioavailability is mainly dependent on chemical phenolic structure but it can also be influenced by the characteristics of the subject (age, gender, genetic profile, etc.), (Teng and Chen, 2019).

As evidenced by box plot (Figure 3), the inter-individual variability in plasma metabolites was quite high. Moreover, after capsule ingestion, the analysis of plasma isolated from few volunteers indicated the presence of traces of other metabolites (e.g., gallic acid and dihydroxyferulic acid glucuronide) (data not shown). Although, *Hamamelis v.* is largely used as folk medicine, data on the possible active constituents is missing. As far as we know, no report has been published on the presence of galloyl derivative metabolites in plasma after *Hamamelis v.* consumption. The only data on metabolites of gallotannin-derivatives comes from studies on mango that is a rich source of these compounds. Three studies reported the presence of 4-O-methylgallic acid, 4-O-methylgallic acid-3-O-sulfate, pyrogallol-O-sulfate, methylpyrogallol-O-sulfate, and catechol-O- sulfate in human plasma after post-prandial consumption of 400 g of mango pulp (Fang et al., 2018; Barnes et al., 2019; Barnes et al., 2020). On the other hand, Barnes *et al.* did not detect any metabolites in plasma after 10 days of mango pulp consumption (400 g/day) because the concentration was too low for the detection in fasting plasma (seven metabolites were identified in urines) (Barnes et al., 2016). In one of his studies, Barnes speculated that the great interindividual variability in the plasmatic concentration of gallotannin metabolites could be ascribed to BMI, microbiota composition and exposure time to gallotannions of the subjects (Barnes et al., 2019).

### Extracellular Matrix Expression in Human Dermal Fibroblast

In photoaged skin, major alterations are seen in dermal connective tissue, characterized by damaged and disorganized ECM network (Yamaba et al., 2016). The ECM mainly consists of the fibrous proteins, collagen and elastin, and of the associated-microfibrils, fibronectin and laminin, which are surrounded by a viscoelastic gel made of polymers of proteoglycans (Tracy et al., 2016). Collagen, which is the main structural element of the ECM, is predominantly transcribed and secreted by fibroblasts (Frantz et al., 2010), that are the major components of the dermis and one of the most important players participating in the metabolism of ECM proteins.

**FIGURE 4:**
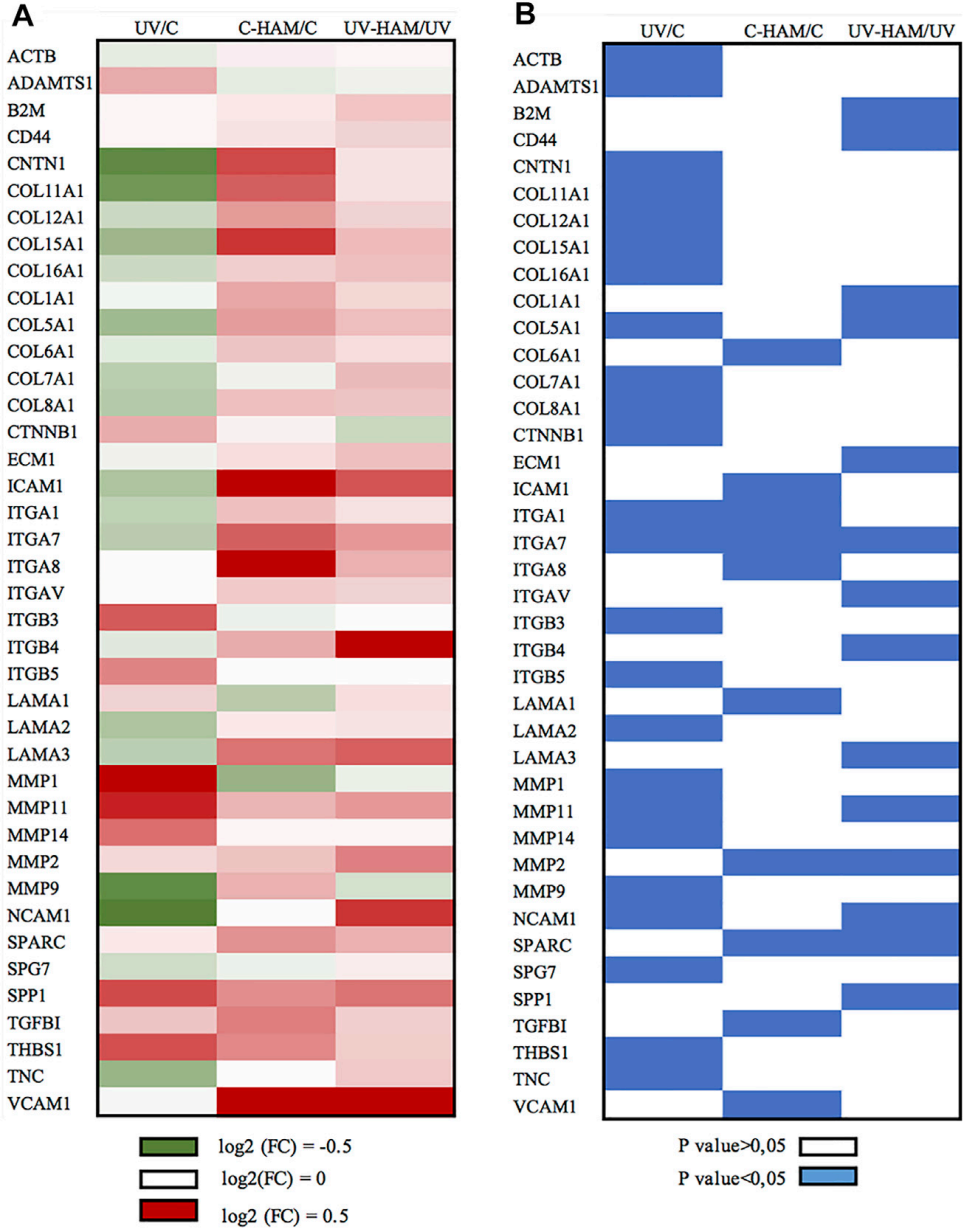
Graphical presentation of (A) log (2) Fold Change (FC) mean of gene expression and (B) the corresponding *p* value evaluated by paired *t*-test. **(A)** Green cells represent the downregulation at the interval 0–0.5 log (2) FC, white cells indicate no changes and red cells represent upregulation at the interval 0–0.5 log (2) FC. **(B)** blue cells indicate the statistically significant genes (*p* < 0.05). The figure shows all the genes that are significantly regulated at least in one treatment.

As shown in Figure 4B (UV/C column), UV-A exposure of HDF cells incubated with serum time 0 (UV) significantly modulated the expression of 24 genes compared to control (C). Among these, 16 were downregulated and 8 upregulated. Among the downregulated genes, 7 coding for collagen proteins (*COL11A1, COL12A1, COL15A1, COL16A1, COL5A1, COL7A1 and COL8A1*), the other downregulated genes were: *CNTN1, ITGA1, ITGA7, LAMA2, NCAM1, SPG7, TNC* and ACTB. On the contrary, the metalloproteinases genes *MMP-1, MMP-11* and *MMP-14* were upregulated, with the exception of *MMP-9* that resulted downregulated. Finally, the other upregulated genes were: *ADAMTS1, CTNNB1, ITGB3, ITGB5 and THBS1* (Figure 4A). This pattern of gene regulation is consistent with the well-known harmful effects of UV-A on dermal fibroblasts (Marionnet et al., 2012; Quan et al., 2013; Yamaba et al., 2016).

**FIGURE 5:**
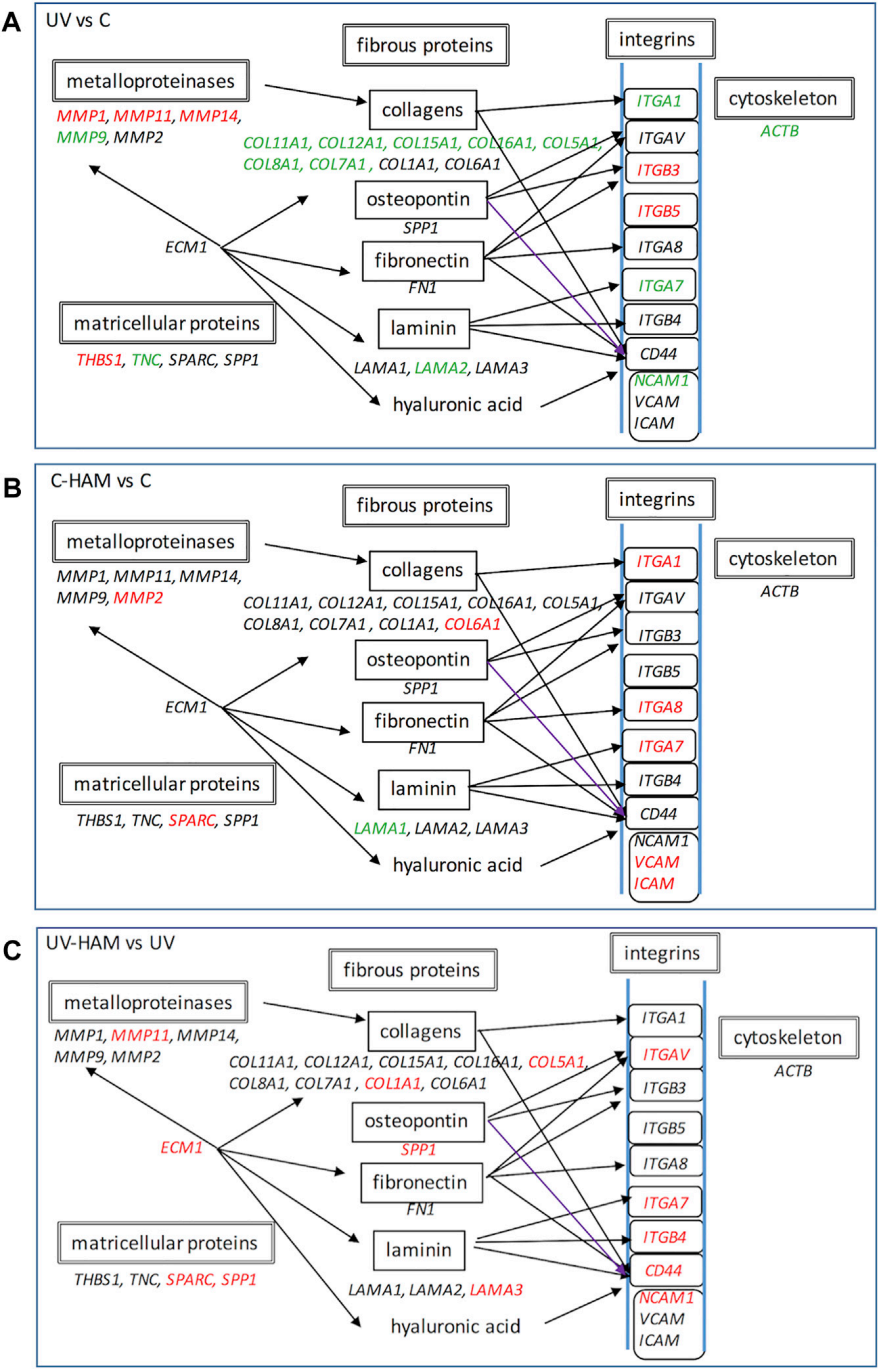
Schematic representation of gene expression regulation of UV treatment with respect to control **(A)**, C-HAM with respect to control **(B)** and UV-HAM with respect to UV treatment **(C)**. Names in Italic font indicates genes which expression is upregulated (in red), downregulated (in green) and statistically not significant (in black). Modified by KEGG pathway (https://www.genome.jp/dbget-bin/www_bget?pathway:map04512).

In fact, as shown in Figure 5A, UV-A irradiation affected the expression of ECM genes, compromising the organization of overall ECM network. We observed a downregulation of genes coding for important structural elements such as the minor fibrillar collagen (COL11A1 and COL16A1) and the fibril-forming collagen COL5A1, but also laminin 2 (LAMA2), fibronectin (FN1) (at limit of statistical significance, *p* = 0.056) (Supplementary Table S2), integrin 1 (ITGA1), integrin 7 (ITGA7) and the nonfibrillar collagens COL7A1 and COL6A1 (at limit of statistical significance, *p* = 0.052) (Supplementary Table S2), that compose a reticular net and connect cells to the basement membrane (Tracy et al., 2016). Integrins are a major class of receptors that serve to connect fibroblast to ECM and act as signalling receptors (Barczyk et al., 2010). Noteworthy, UV-A radiation also induced a significant decrease of actin B (ACTB) gene expression, coding for one of the main cytoskeletal filaments, which is consistent with the collapsed cell shape observed in photodamaged fibroblasts (Yamaba et al., 2016).

Beyond the downregulation of the structural ECM component genes, UV-A irradiation induced the expression of some matrix metalloproteinases genes. MMPs are a family of 24 zinc dependent proteolytic enzymes responsible of degrading ECM proteins. It is known that acute exposure to UV-A radiation induces gene expression of MMP-1, MMP-3, and MMP-9 that leads to collagen fragmentation (Quan et al., 2009; Marionnet et al., 2012). Accordingly, UV-A exposure significantly upregulated MMP-1, MMP-11 and MMP-14, while MMP-3 upregulation was at the limit of statistical significance (*p* = 0.054) (Supplementary Table S2). On the other hand, MMP-9 was significantly downregulated while ADAMTS1, another important proteolytic protein that plays a role in remodelling ECM (de Arao Tan et al., 2013), was significantly upregulated. The genes coding the tissue inhibitor of MMPs, TIMP1, 2 and 3 were, instead, not regulated (Supplementary Table S2).

Finally, UV-A irradiation induced the upregulation of the genes coding for integrins ITGB3 and ITGB5, whose ligands are fibronectin and vitronectin; ITGB3 has been associated to fibroblast senescence by activating the transforming growth factor β (TGF-β) pathway and then starting the healing process (Rapisarda et al., 2017). UV-A irradiation also modulated the expression of genes coding for matricellular proteins, i.e. thrombospondin (THBS-1), osteonectin (SPARC, the secreted protein acidic and rich in cysteine) and osteopontin (SPP1), even if only the former was significantly upregulated, and downregulated tenascin (TNC). Matricellular proteins have functions in tissue remodelling and repair (Sodek et al., 2002; Mosher & Adams, 2012). In particular, TNC, THBS1 and SPARC show high level of expression in response to injury and induce de-adhesion, characterized by disruption of focal adhesions and reorganization of actin stress fibers (Bornstein, 2009), whereas SPP1 interacts with collagen and fibronectin (Mukherjee et al., 1995) and contains several cell adhesive domains that interact with integrins and CD44, a cell surface adhesion receptor that links several ECM proteins (Sodek et al., 2002).

In the C-HAM treatment, HDF cells were incubated with serum containing the metabolites of *Hamaforton™*; since metabolites had a total concentration in serum of around 1.5 μM and serum was added to the medium at a concentration of 10%, the final concentration of metabolites in cell culture medium was around 150 nM. To evaluate if *Hamaforton™* metabolites affected ECM gene transcription, we compared the gene modulatory effects of C-HAM with C treatments. Interestingly, the serum containing *Hamaforton™* metabolites significantly affected gene transcription; precisely, 10 genes were significantly modulated when compared to control (C): COL6A1, ITGA1, ITGA7, ITGA8, MMP-2, SPARC, TGFBI, VCAM1 and ICAM1 (COL5A1 was at the limit of statistical significance with *p* = 0.05), were upregulated while LAMA1 was downregulated (Figure 4, C-HAM/C column) (Figure 5B).

In order to confirm that the effects on gene expression were effectively related to *Hamaforton™* metabolites, we also analysed the effects of serum isolated after placebo supplementation (C-Pla treatment) in respect to its control. None of the 10 genes was differently regulated in C-Pla (data not shown).

The up-regulation of metalloproteinase as well as collagens, integrins and cell adhesion genes in absence of specific harmful injury suggests that *Hamaforton™* metabolites are able to activate an adaptive cellular stress response pathway, as demonstrated in different studies (Son et al., 2008; Calabrese and Mattson, 2017). According to this principle, *Hamaforton™* metabolites would induce a mild stress that could “preconditionate” fibroblast cells to the following ultraviolet irradiation stress with a potential protective effect.

Noteworthy is the upregulation of SPARC and of its downstream target TGFBI (Tumbarello et al., 2016). As mentioned above, SPARC is a matricellular protein with homeostasis–regulatory roles in ECM metabolism and function (Ghanemi et al., 2020). SPARC is induced by transforming growth factor-beta 1 (TGF-β1), a key regulator of pro-fibrotic processes, with important roles in fibroblast differentiation and proliferation and wound healing (Carvalheiro et al., 2020). The expression of SPARC is restricted to sites of ECM turnover and increases in response to injury or tissue remodelling probably to facilitate the ECM reorganization for cell migration, proliferation and differentiation, possibly through MMPs regulation (Bradshaw & Sage, 2001). Moreover, the *Hamaforton™* -induced activation of the expression of VCAM and ICAM, important cell adhesion molecules and regulators of leukocyte trafficking, is consistent with the ability of *Hamaforton™* metabolites to induce an adaptive molecular phenotype. In fact, this kind of process is characterized by simultaneous stimulation of many independent cellular functions (Calabrese and Mattson, 2017). Taken together these results show that the metabolites of *Hamaforton™*, despite the very low concentration, were able to differently modulate several genes of collagens, matricellular proteins and membrane receptors that are involved in structure organization and cellular signalling.

The possibility that *Hamaforton™* metabolites could affect UV-A dependent gene expression was evaluated through a comparison between UV-HAM and UV treatments. Among the 24 genes significantly regulated by UV-A, 20 (*COL11A1, COL12A1, COL15A1, COL16A1, COL8A1, COL7A1, MMP-1, MMP-14, MMP-9, ITGA1, ITGB3, ITGB5, TNC, CTNNB1, CNTN1, THBS1, LAMA2, ACTB, ADAMTS1, SPG7*) were not significantly modified by UV-HAM, while the remaining 4 (*NCAM1, COL5A1, MMP-11* and *ITGA7*) were significantly upregulated in UV-HAM with respect to UV treatment (Figure 4, UV-HAM/UV column) (Figure 5C). Among these four, MMP-11 expression was up-regulated, but to a significantly higher extent compared to UV treatment. MMP-11 is unique from a functional point of view as it does not cleave major components of the ECM, contributing very little to the UV-A damage of collagen (Dali-Youcef et al., 2016). Moreover, incubation with *Hamaforton™* metabolites upregulated also the expression of *COL5A1, NCAM1* and *ITGA7* with respect to UV treatment*.* But while *COL5A1* and *NCAM1* were still significantly downregulated in comparison to control (*p* = 0.014 and *p* = 0.040, respectively) *ITGA7* expression was not significantly modified from control (*p* = 0.352).

Additionally, we found 10 more genes differentially expressed in UV-HAM treatment, that were not regulated in UV treatment. Noteworthy, all of them (CD44, COL1A1, ECM1, ITGAV, ITGB4, LAMA3, MMP-2, SPARC, B2M and SPP1) were upregulated with respect to UV treatment (Figure 4A). To confirm that the observed modulation of gene expression was effectively related to the metabolites of *Hamaforton™*, we analysed the effects of serum isolated after placebo supplementation in UV-Pla treatment with respect to UV treatment (data not shown). No genes were differently regulated in the two treatments, with the exception of a significant upregulation of MMP-2 (*p* = 0.039). Among the genes specifically upregulated by the UV-HAM treatment, COL1A1 gene produces the α1(I) component of type I collagen, the most abundant collagen in the human body. ECM1, instead, which was also upregulated compared to UV, is a multifunctional protein that interacts with the majority of other ECM proteins (MMP-9, laminin, collagen type IV and fibronectin) and has a role in maintaining skin integrity (Sercu et al., 2009) (Figure 5C). Interestingly, a photoprotective role has been suggested for this protein based on the observation that patients with lipoid proteinosis (a rare autosomal recessive disorder caused by a mutation in ECM1 gene), who live in sunny regions have more severe phenotype compared to patients in less sunny countries (Van Hougenhouck-Tulleken et al., 2004). CD44, also upregulated in UV-HAM compared to UV treatment, is a ubiquitous hyaluronic acid (HA) receptor that spans the cell plasma membrane (Dicker et al., 2014). While HA plays an important role in organizing the ECM structure, CD44 functions as regulator of the maintenance of HA homeostasis and keratinocytes proliferation (Kaya et al., 1997). It has been shown that UV-A irradiation of keratinocytes significantly decreased the expressions of CD44 and HA (Calikoglu et al., 2006). Therefore, strategies aiming at inhibiting the decrease of CD44 and HA by UV irradiation have been suggested to be useful to prevent the deleterious effects of solar ultraviolet radiations to the skin (Calikoglu et al., 2006). In our experimental model, UV-A exposure did not significantly modulate the expression of CD44 in fibroblasts (Supplementary Table S2), but the expression could have returned to control levels after the 24 h of recovery (Calikoglu et al., 2006). Nevertheless, the presence of *Hamaforton™* metabolites significantly upregulated the expression of CD44 with respect to both UV treatment and control (*p* = 0.037) (Supplementary Table S2). As we mentioned above, SPP1 and SPARC are matricellular proteins involved in skin remodelling and repair. SPP1 can also act also as structural protein by binding the integrin ITGAV (Singh et al., 2010) but also the receptor CD44 (Weber et al., 1996), both of which were up-regulated in presence of *Hamaforton™* metabolites. While SPARC, which was also upregulated in C-HAM treatment, plays a critical role in collagen assembly and deposition. The upregulation of all these genes, together with genes like COL1A1, COL5A1 and LAMA3 that encodes for structural proteins of ECM, strongly suggests that *Hamaforton™* metabolites could influence the recovery process following UV-A damage.

## Conclusion

The extracellular matrix is an active and complex tissue component that beyond simply anchoring cells is capable of influencing cell proliferation, adhesion and migration, and regulates cell differentiation and death. ECM composition is highly heterogeneous and dynamic, being constantly remodeled and modulated and its remodeling is involved in the regulation of cell differentiation, branching morphogenesis, angiogenesis, bone remodeling, and wound repair. UV-A irradiation of fibroblasts was shown to induce anomalous ECM dynamics through the deregulation of important gene expression. In our experimental model, the presence of *Hamaforton™* metabolites did not affect the expression of most of the gene expression changes induced by UV-A but, despite their very low concentration, they were able to activate the expression of 10 different genes involved in diverse repair processes fundamental for the maintenance of skin integrity. To our knowledge, this is the first study that demonstrates bioavailability of *Hamaforton™* phenolic compounds by measuring the kinetics of appearance of galloyl derivatives in plasma, as well as the effects of these metabolites on cultured fibroblast ECM gene expression. Obviously, further studies are necessary to explore the mechanism of action of the metabolites on the modulation of cell response to UV-A irradiation.

## Data Availability

The original contributions presented in the study are included in the article/Supplementary Materials, further inquiries can be directed to the corresponding author/s.

## Abbreviations

ECM, Extracellular Matrix; HDF, Human adult Dermal Fibroblast; UV-A, Ultraviolet A; UV-B, Ultraviolet B. Cellular treatments; C, control cells incubated with time 0 serum; C-HAM, cells incubated with human serum isolated 3 hours after *Hamaforton™* supplementation; C-Pla, cells incubated with human serum isolated 3 hours after placebo supplementation; UV, cells incubated with time 0 serum and exposed to UV-A; UV-HAM, cells incubated with human serum isolated 3 hours after *Hamaforton™* supplementation and exposed to UV-A; UV-Pla, cells incubated with human serum isolated 3 hours after placebo supplementation and exposed to UV-A.
