# Supplementary material for: Human Metabolites of Hamaforton™ (Hamamelis virginiana L. Extract) Modulates Fibroblast Extracellular Matrix Components in Response to UV-A Irradiation
Source: Front Pharmacol. 2021 Dec 17;12:747638. doi: 10.3389/fphar.2021.747638 (PMC8719534; doi:10.3389/fphar.2021.747638)
Supplement: Supplementary file 1 [file Table1.pdf]

Metabolite	Standard for quantification	MRM	Declustering Potential (V)	Collision Energy (eV)
4-O-methyl gallic acid (4OMG)	4OMG	183>168	-30	-15
4-O-methyl gallic acid sulphate	4OMG	263>183	-30	-15
trimethyl gallic acid glucuronide	4OMG	387>211	-30	-15

Supplementary Table S1: MRM condition and transitions of identified metabolites
