# Supplementary material for: Human Metabolites of Hamaforton™ (Hamamelis virginiana L. Extract) Modulates Fibroblast Extracellular Matrix Components in Response to UV-A Irradiation
Source: Front Pharmacol. 2021 Dec 17;12:747638. doi: 10.3389/fphar.2021.747638 (PMC8719534; doi:10.3389/fphar.2021.747638)
Supplement: Supplementary file 2 [file Table2.pdf]

								p value			
**Unigene**	**Refseq**		**Description**	C	C-HAM	UV	UV-HAM	UV vs C	UV-HAM vs UV	C-HAM vs C	UV-HAM vs C
Hs.643357	NM_006988	ADAMTS1	ADAM metallopeptidase with thrombospondin type 1 motif, 1	0.02808	0.02619	0.03122	0.03004	0.03862	0.33542	0.29007	0.20166
Hs.131433	NM_139025	ADAMTS13	ADAM metallopeptidase with thrombospondin type 1 motif, 13	0.00009	0.00009	0.00008	0.0001	0.18186	0.12479	0.84106	0.45432
Hs.271605	NM_007037	ADAMTS8	ADAM metallopeptidase with thrombospondin type 1 motif, 8	0.00014	0.00013	0.0001	0.00012	0.36656	0.34148	0.79995	0.77841
Hs.502328	NM_000610	CD44	CD44 molecule (Indian blood group)	0.18397	0.1905	0.18611	0.19718	0.65081	0.01842	0.29211	0.03786
Hs.461086	NM_004360	CDH1	Cadherin 1, type 1, E-cadherin (epithelial)	0.00005	0.00004	0.00004	0.00005	0.58295	0.5875	0.52305	0.87809
Hs.476092	NM_003278	CLEC3B	C-type lectin domain family 3, member B	0.00607	0.00673	0.00565	0.00604	0.18696	0.15767	0.08924	0.90005
Hs.143434	NM_001843	CNTN1	Contactin 1	0.00017	0.0002	0.00012	0.00012	0.00676	0.75991	0.39263	0.01917
Hs.523446	NM_080629	COL11A1	Collagen, type XI, alpha 1	0.00107	0.00123	0.00075	0.00076	0.01752	0.88251	0.26591	0.02638
Hs.101302	NM_004370	COL12A1	Collagen, type XII, alpha 1	0.00483	0.0054	0.00429	0.00458	0.04795	0.10762	0.20844	0.34308
Hs.409662	NM_021110	COL14A1	Collagen, type XIV, alpha 1	0.00011	0.00013	0.00007	0.00008	0.14946	0.43698	0.6127	0.20222
Hs.409034	NM_001855	COL15A1	Collagen, type XV, alpha 1	0.00017	0.00021	0.00013	0.00013	0.04842	0.96163	0.1026	0.11608
Hs.368921	NM_001856	COL16A1	Collagen, type XVI, alpha 1	0.00705	0.00756	0.00638	0.00691	0.00201	0.14917	0.14721	0.66486
Hs.172928	NM_000088	COL1A1	Collagen, type I, alpha 1	0.92128	1.02749	0.89105	0.93825	0.43534	0.01807	0.0755	0.64437
Hs.508716	NM_001846	COL4A2	Collagen, type IV, alpha 2	0.0225	0.02322	0.02337	0.02419	0.16221	0.38371	0.39652	0.09453
Hs.210283	NM_000093	COL5A1	Collagen, type V, alpha 1	0.02365	0.02675	0.01951	0.02124	0.00145	0.02522	0.05005	0.01397
Hs.474053	NM_001848	COL6A1	Collagen, type VI, alpha 1	0.42813	0.46263	0.40571	0.42306	0.05206	0.243	0.03967	0.54529
Hs.420269	NM_001849	COL6A2	Collagen, type VI, alpha 2	0.46721	0.50761	0.45127	0.47042	0.25258	0.23545	0.05708	0.86997
Hs.476218	NM_000094	COL7A1	Collagen, type VII, alpha 1	0.00576	0.00553	0.00505	0.0054	0.00003	0.37097	0.57341	0.43226
Hs.654548	NM_001850	COL8A1	Collagen, type VIII, alpha 1	0.00354	0.0038	0.00303	0.00325	0.01785	0.27394	0.26688	0.05495
Hs.410037	NM_001901	CTGF	Connective tissue growth factor	0.03867	0.03783	0.0416	0.04151	0.18996	0.91465	0.68698	0.11659
Hs.656653	NM_001903	CTNNA1	Catenin (cadherin-associated protein), alpha 1, 102kDa	0.04738	0.04859	0.04832	0.04898	0.55064	0.48573	0.45073	0.40857
Hs.712929	NM_001904	CTNNB1	Catenin (cadherin-associated protein), beta 1, 88kDa	0.01256	0.01276	0.01402	0.01254	0.00602	0.07929	0.71124	0.98449
Hs.166011	NM_001331	CTNND1	Catenin (cadherin-associated protein), delta 1	0.01412	0.01394	0.01413	0.01418	0.9863	0.84587	0.7312	0.88218
Hs.314543	NM_001332	CTNND2	Catenin (cadherin-associated protein), delta 2 (neural plakophilin-related arm-repeat protein)	0.00009	0.00008	0.00008	0.00005	0.06651	0.00515	0.37969	0.00234
Hs.81071	NM_004425	ECM1	Extracellular matrix protein 1	0.01217	0.0127	0.01177	0.01275	0.49361	0.02156	0.27494	0.40919
Hs.203717	NM_002026	FN1	Fibronectin 1	3.92858	4.2862	3.66583	3.84101	0.05641	0.13473	0.15145	0.48615
Hs.57697	NM_001523	HAS1	Hyaluronan synthase 1	0.00076	0.00054	0.00073	0.00066	0.55862	0.17411	0.09494	0.23020
Hs.643447	NM_000201	ICAM1	Intercellular adhesion molecule 1	0.00257	0.00375	0.00216	0.00258	0.05787	0.05834	0.02387	0.95982
Hs.644352	NM_181501	ITGA1	Integrin, alpha 1	0.01138	0.01234	0.01007	0.01041	0.01052	0.31003	0.03617	0.02562
Hs.482077	NM_002203	ITGA2	Integrin, alpha 2 (CD49B, alpha 2 subunit of VLA-2 receptor)	0.01957	0.01566	0.02056	0.01962	0.51746	0.45796	0.1314	0.98520
Hs.265829	NM_002204	ITGA3	Integrin, alpha 3 (antigen CD49C, alpha 3 subunit of VLA-3 receptor)	0.07036	0.06857	0.07108	0.0722	0.78418	0.64021	0.55331	0.51259
Hs.440955	NM_000885	ITGA4	Integrin, alpha 4 (antigen CD49D, alpha 4 subunit of VLA-4 receptor)	0.01954	0.02028	0.01991	0.01992	0.35276	0.98364	0.42532	0.44221
Hs.505654	NM_002205	ITGA5	Integrin, alpha 5 (fibronectin receptor, alpha polypeptide)	0.145	0.13846	0.14829	0.14605	0.50035	0.65434	0.33584	0.87481
Hs.133397	NM_000210	ITGA6	Integrin, alpha 6	0.00269	0.00232	0.00233	0.00222	0.07556	0.52507	0.13784	0.01388
Hs.524484	NM_002206	ITGA7	Integrin, alpha 7	0.00233	0.00279	0.00194	0.0022	0.03879	0.02098	0.03025	0.35258
Hs.592472	NM_003638	ITGA8	Integrin, alpha 8	0.00008	0.00011	0.00007	0.00007	0.40162	0.86957	0.01769	0.32000
Hs.174103	NM_002209	ITGAL	Integrin, alpha L (antigen CD11A (p180), lymphocyte function-associated antigen 1; alpha polypeptide)	0.00015	0.0001	0.0001	0.00014	0.46052	0.32006	0.4532	0.86959
Hs.172631	NM_000632	ITGAM	Integrin, alpha M (complement component 3 receptor 3 subunit)	0.0001	0.00006	0.00007	0.0001	0.55125	0.36685	0.48051	0.97465
Hs.436873	NM_002210	ITGAV	Integrin, alpha V (vitronectin receptor, alpha polypeptide, antigen CD51)	0.0267	0.02855	0.02649	0.02802	0.83754	0.04401	0.18146	0.08600
Hs.643813	NM_002211	ITGB1	Integrin, beta 1 (fibronectin receptor, beta polypeptide, antigen CD29 includes MDF2, MSK12)	0.72729	0.74048	0.66995	0.6759	0.08336	0.69081	0.71703	0.13636
Hs.375957	NM_000211	ITGB2	Integrin, beta 2 (complement component 3 receptor 3 and 4 subunit)	0.00006	0.00004	0.00004	0.00005	0.55021	0.12003	0.42093	0.92139
Hs.218040	NM_000212	ITGB3	Integrin, beta 3 (platelet glycoprotein IIIa, antigen CD61)	0.02209	0.02113	0.02707	0.02699	0.00025	0.94523	0.40158	0.00341
Hs.632226	NM_000213	ITGB4	Integrin, beta 4	0.00006	0.00006	0.00005	0.00008	0.30051	0.03231	0.81586	0.15111
Hs.13155	NM_002213	ITGB5	Integrin, beta 5	0.02983	0.02992	0.03512	0.03501	0.0003	0.94761	0.92348	0.01805
Hs.521869	NM_000216	ANOS1	Kallmann syndrome 1 sequence	ND	ND	ND	ND				
Hs.270364	NM_005559	LAMA1	Laminin, alpha 1	0.00032	0.00028	0.00033	0.00033	0.69876	0.81132	0.03498	0.61152
Hs.200841	NM_000426	LAMA2	Laminin, alpha 2	0.0039	0.00396	0.00329	0.00336	0.01609	0.70177	0.77818	0.06479
Hs.436367	NM_000227	LAMA3	Laminin, alpha 3	0.00054	0.00064	0.00047	0.00056	0.0672	0.0208	0.13928	0.57211
Hs.650585	NM_002291	LAMB1	Laminin, beta 1	0.07931	0.07877	0.07418	0.07609	0.11452	0.43647	0.84369	0.22305
Hs.497636	NM_000228	LAMB3	Laminin, beta 3	0.00194	0.00135	0.00151	0.00193	0.55121	0.29777	0.37667	0.99914
Hs.609663	NM_002293	LAMC1	Laminin, gamma 1 (formerly LAMB2)	0.32225	0.35221	0.3187	0.33205	0.81908	0.21105	0.07161	0.44430
Hs.83169	NM_002421	MMP1	Matrix metallopeptidase 1 (interstitial collagenase)	0.03259	0.02268	0.05469	0.04833	0.00019	0.33644	0.09047	0.00669
Hs.2258	NM_002425	MMP10	Matrix metallopeptidase 10 (stromelysin 2)	0.00022	0.00013	0.00017	0.00025	0.59229	0.15854	0.29905	0.78325
Hs.143751	NM_005940	MMP11	Matrix metallopeptidase 11 (stromelysin 3)	0.00027	0.0003	0.00035	0.0004	0.00203	0.02451	0.34651	0.00060
Hs.1695	NM_002426	MMP12	Matrix metallopeptidase 12 (macrophage elastase)	0.13324	0.12743	0.17424	0.11334	0.47741	0.4413	0.92643	0.79896
Hs.2936	NM_002427	MMP13	Matrix metallopeptidase 13 (collagenase 3)	ND	ND	ND	ND				
Hs.2399	NM_004995	MMP14	Matrix metallopeptidase 14 (membrane-inserted)	0.03739	0.0358	0.04359	0.04318	0.01557	0.91822	0.54826	0.20905
Hs.80343	NM_002428	MMP15	Matrix metallopeptidase 15 (membrane-inserted)	0.00066	0.00041	0.00043	0.00052	0.50457	0.39176	0.44233	0.70155
Hs.546267	NM_005941	MMP16	Matrix metallopeptidase 16 (membrane-inserted)	0.00462	0.00449	0.00453	0.0046	0.67195	0.75484	0.6654	0.94856
Hs.513617	NM_004530	MMP2	Matrix metallopeptidase 2 (gelatinase A, 72kDa gelatinase, 72kDa type IV collagenase)	0.22974	0.24959	0.24212	0.28262	0.18306	0.03988	0.03908	0.00139
Hs.375129	NM_002422	MMP3	Matrix metallopeptidase 3 (stromelysin 1, progelatinase)	0.12583	0.10869	0.14593	0.1462	0.05395	0.98262	0.29475	0.17245
Hs.2256	NM_002423	MMP7	Matrix metallopeptidase 7 (matrilysin, uterine)	0.00002	0.00002	0.00001	0.00002	0.09885	0.06493	0.30591	0.42679
Hs.161839	NM_002424	MMP8	Matrix metallopeptidase 8 (neutrophil collagenase)	0.00021	0.00011	0.00013	0.0002	0.50544	0.37106	0.3601	0.95458
Hs.297413	NM_004994	MMP9	Matrix metallopeptidase 9 (gelatinase B, 92kDa gelatinase, 92kDa type IV collagenase)	0.00003	0.00003	0.00002	0.00002	0.02406	0.33779	0.886	0.01839
Hs.503878	NM_000615	NCAM1	Neural cell adhesion molecule 1	0.00033	0.00032	0.00021	0.00027	0.0002	0.0167	0.81849	0.04084
Hs.514412	NM_000442	PECAM1	Platelet/endothelial cell adhesion molecule	0.00032	0.00028	0.00026	0.00031	0.46827	0.47505	0.52615	0.95456
Hs.82848	NM_000450	SELE	Selectin E	0.00004	0.00002	0.00002	0.00004	0.442	0.34419	0.40617	0.90930
Hs.728756	NM_000655	SELL	Selectin L	0.00009	0.00006	0.00006	0.00008	0.53616	0.30632	0.53785	0.89059
Hs.73800	NM_003005	SELP	Selectin P (granule membrane protein 140kDa, antigen CD62)	ND	ND	ND	ND				
Hs.371199	NM_003919	SGCE	Sarcoglycan, epsilon	0.01397	0.01495	0.01279	0.01338	0.10283	0.30583	0.17254	0.55885
Hs.111779	NM_003118	SPARC	Secreted protein, acidic, cysteine-rich (osteonectin)	0.46351	0.53538	0.4746	0.52323	0.5002	0.02883	0.00093	0.02906
Hs.185597	NM_003119	SPG7	Spastic paraplegia 7 (pure and complicated autosomal recessive)	0.0092	0.00886	0.0083	0.00847	0.04015	0.49169	0.25828	0.09882
Hs.313	NM_000582	SPP1	Secreted phosphoprotein 1	0.00035	0.00038	0.00042	0.0005	0.08643	0.02592	0.49471	0.00192
Hs.369397	NM_000358	TGFBI	Transforming growth factor, beta-induced, 68kDa	0.60628	0.72007	0.64928	0.68922	0.17741	0.12869	0.00937	0.00444
Hs.732539	NM_003246	THBS1	Thrombospondin 1	0.11698	0.13673	0.15024	0.15937	0.01559	0.10071	0.08305	0.00557
Hs.371147	NM_003247	THBS2	Thrombospondin 2	0.03695	0.04017	0.03689	0.03962	0.93108	0.09152	0.1342	0.06228
Hs.169875	NM_007112	THBS3	Thrombospondin 3	0.00049	0.00054	0.00051	0.00056	0.41277	0.09331	0.32598	0.04711
Hs.522632	NM_003254	TIMP1	TIMP metallopeptidase inhibitor 1	0.16296	0.16256	0.16175	0.15921	0.76482	0.552	0.94561	0.58869
Hs.633514	NM_003255	TIMP2	TIMP metallopeptidase inhibitor 2	0.07737	0.08521	0.08242	0.08272	0.16106	0.95129	0.11141	0.18192
Hs.644633	NM_000362	TIMP3	TIMP metallopeptidase inhibitor 3	0.04406	0.045	0.04302	0.0425	0.49491	0.78461	0.52718	0.33233
Hs.143250	NM_002160	TNC	Tenascin C	0.05114	0.05123	0.04185	0.04457	0.00134	0.15063	0.96275	0.01527
Hs.109225	NM_001078	VCAM1	Vascular cell adhesion molecule 1	0.00025	0.00037	0.00024	0.0003	0.5323	0.15298	0.0135	0.10963
Hs.643801	NM_004385	VCAN	Versican	0.00867	0.00856	0.00977	0.00937	0.18638	0.51195	0.88544	0.17600
Hs.2257	NM_000638	VTN	Vitronectin	0.00009	0.00009	0.00006	0.00007	0.12781	0.41566	0.96817	0.51032
Hs.520640	NM_001101	ACTB	Actin, beta	1.82481	1.85693	1.7365	1.76053	0.03391	0.4811	0.5607	0.14282
Hs.534255	NM_004048	B2M	Beta-2-microglobulin	0.47531	0.48847	0.47965	0.51595	0.81812	0.0192	0.5732	0.08158

Supplementary Table S2. List of all the gene expressions in alphabetical order analysed with RT² Profiler™ PCR Array (Qiagen) expressed as average of 2^-ΔCt for each treatment. P values were calculated with paired t-test. Differences with *P* values < 0.05 were considered significant.
